# The Purification and Characterization of a Novel Neutral Protease from *Volvariella volvacea* Fruiting Bodies and the Enzymatic Digestion of Soybean Isolates

**DOI:** 10.3390/jof11030190

**Published:** 2025-03-01

**Authors:** Baoting Xu, Zhiping Li, Qian Guo, Lei Zha, Chuanhua Li, Panling Yu, Mingjie Chen, Yan Zhao

**Affiliations:** 1 College of Food Science and Technology, Shanghai Ocean University, Shanghai 201306, China; xbting0919@163.com (B.X.); yupanling13@163.com (P.Y.); 2 Institute of Edible Fungi, Shanghai Academy of Agricultural Sciences, Shanghai 201403, China; qalizhiping@163.com (Z.L.); guoqian@saas.sh.cn (Q.G.); zhalei@saas.sh.cn (L.Z.); 15221098780@163.com (C.L.)

**Keywords:** *Volvariella volvacea*, proteases, purification, soybean-isolate protein

## Abstract

A novel protease was isolated from the fruiting bodies of the straw mushroom *Volvariella volvacea*. The protease was purified 13.48-fold using a series of techniques, including ammonium sulfate precipitation, ultrafiltration, diethylaminoethyl fast-flow (DEAE FF) ion-exchange chromatography, and Superdex 75 gel filtration chromatography, resulting in a specific enzyme activity of 286.82 U/mg toward casein as a substrate. Sodium dodecyl sulfate–polyacrylamide gel electrophoresis (SDS-PAGE) analysis revealed that the purified protease had a molecular weight of 24 kDa. The enzyme exhibited optimal activity at pH 7 and 50 °C, showing sensitivity to alkaline conditions and instability at elevated temperatures. The presence of Ca^2+^ significantly enhanced enzyme activity, whereas Ni^2+^ and Cu^2+^ exerted strong inhibitory effects, with other metal ions showing weak inhibition. β-mercaptoethanol, Tween-80, and Triton X-100 had more pronounced inhibitory effects, whereas PMSF, EDTA, and CTAB had weaker inhibitory effects. The Michaelis constant (Km) and maximum velocity (Vm) of the protease were determined to be 1.34 g/L and 3.45 μg/(mL·min), respectively. The protease exhibited a greater degree of enzymatic degradation of soybean-isolate protein (7.58%) compared to trypsin (5.24%), with the enzyme product containing a high percentage of medicinal amino acids (73.54%), particularly phenylalanine (Phe) and arginine (Arg), suggesting their presence at the enzyme’s active site. These findings suggest that the protease from *V. volvacea* holds promising potential for applications in the food industry, particularly in protein hydrolysate production and flavor enhancement.

## 1. Introduction

Proteases possess several advantages, such as mild reaction conditions, high catalytic efficiency, and specificity, making them widely utilized in various industries, including food, laundry, leather, and pharmaceuticals. Despite their extensive use in industrial production, there is a pressing need to develop novel proteases in numerous industrial sectors. Consequently, the discovery of new proteases is a vital research direction. Many fungal species can secrete and produce proteases in nature. These fungi exhibit distinct characteristics due to their respective living environments, resulting in their proteases having varying properties, including optimal reaction temperatures and pH levels [1]. For example, a protease purified from antler fungi demonstrated the highest enzyme activity at pH 6.8 and a temperature of 36 °C [2], while Majumder et al. [3] purified an acidic protease from chickweed fungi, which exhibited maximum enzyme activity at 45 °C. Fungi are capable of secreting and synthesizing a multitude of proteases, exemplifying the diversity of fungal proteases. In downstream engineering applications, fungal proteases have a broader range of uses than bacterial proteases due to their superior characteristics. As a result, fungal proteases occupy a significant position in protease preparations. As valuable protein sources, edible fungi represent crucial sources of proteases, and numerous researchers have focused their efforts on investigating edible fungal proteases.

Several studies have reported the discovery of novel proteases derived from edible mushrooms. These include a serine protease isolated from *Hypsizigus marmoreus* [4] and a metalloprotease derived from *Laccocephalum mylittae* [5]. Consequently, to obtain a greater variety of fungal proteases, edible mushrooms have also been the subject of interest for many researchers, given that they are rich in numerous active ingredients, including proteins, sterols, polysaccharides, triterpenoids, and nucleosides, among others [6]. Most researchers domestically and internationally have focused on the functional constituents of edible mushrooms, particularly edible fungal polysaccharides and triterpenoids. Polysaccharides are the primary research focus in this area, with most studies being low-level repetitive research [7]. Edible mushroom protein is an essential component of these mushrooms, which have a higher protein content than most other plants. Previous studies have focused primarily on the antioxidant and immune-enhancing properties of edible mushroom proteins, whereas studies on proteases in edible mushrooms are relatively limited. This makes the study of edible mushroom proteases a promising area of research.

Our study aimed to isolate and characterize a novel protease from *Volvariella volvacea*, evaluate its biochemical and kinetic properties, and assess its potential industrial applications. In the present study, the fruiting bodies of *V. volvacea* were stored at 4 °C for 36 h to induce autolysis. The material was subsequently used to extract proteases from *V. volvacea* for further characterization. The crude enzyme mixture containing the protease was then separated and purified to obtain a purer form of the *V. volvacea* protease, and its enzymatic properties and functions were initially investigated. In addition, we compared its soybean protein digestion profile (peptide distribution and free-amino-acid release) with that of trypsin, a well-characterized commercial protease. This work aims to expand the repertoire of fungal proteases for specialized biocatalytic applications while providing insights into the relationship between *V. volvacea* protease activity and postharvest autolysis.

## 2. Materials and Methods

### 2.1. Mushroom Material

The fruiting bodies of *Volvariella volvacea* were obtained from the Institute of Edible Fungi, Shanghai Academy of Agricultural Sciences.

### 2.2. Main Reagents and Instruments

The casein protein and Folin reagents were purchased from Sigma (Kawasaki, Japan); L-tyrosine was sourced from China National Pharmaceutical Group Co., Ltd., (Beijing, China); and the bicinchoninic acid (BCA) protein assay kit was obtained from Beyotime Biotechnology (Shanghai, China). The diethylaminoethyl fast-flow (DEAE FF) ion-exchange column (1 cm × 5 cm) and Superdex 75 Increase 10/300 gel filtration column (1 cm × 12.5 cm) were purchased from General Electric Company (Boston, MA, USA); the UV-1800 UV spectrophotometer was obtained from Shimadzu Instruments Suzhou Co., Ltd. (Suzhou, China). Homogenization was performed using a Polytron PT 2500E homogenizer (Kinematica, Littau, Switzerland). Electrophoresis was performed using a Mini-PROTEAN Tetra System (Bio-Rad, Hercules, CA, USA). Protein purification was conducted using an ÄKTA pure system (GE Healthcare, Chicago, IL, USA). Amino acid analysis was performed using an L-8900 amino acid analyzer (Hitachi, Tokyo, Japan).

### 2.3. Extraction of Crude Enzyme

Fresh fruiting bodies (100 g) were stored at 4 °C for 36 h to induce autolysis and then homogenized in distilled water (1:10 *w*/*v*) using a Polytron PT 2500E homogenizer (Kinematica, Switzerland). The homogenate was kept at 4 °C for 2 h, followed by centrifugation at 5000 rpm for 10 min (4 °C). The supernatant was collected as the crude enzyme mixture.

### 2.4. Determination of Protease Activity and Protein Content

Protease activity was determined using casein as the substrate according to the methods of Zheng et al. [8], with some modifications. The enzymatic reaction was carried out at pH 7.0 in 20 mM phosphate buffer. Briefly, the enzyme mixture (200 μL) was mixed with 200 μL of casein solution (prepared in 50 mM phosphate buffer, pH 7.0) and incubated at 40 °C for 10 min. The reaction was then terminated by adding 400 μL of 0.4 M trichloroacetic acid (TCA) and centrifuging at 8000 r/min for 10 min. Two hundred microlitres of supernatant, 1 mL sodium carbonate solution (0.4 M), and 200 μL of Folin’s reagent were quickly mixed. Then, the samples were incubated at 40 °C for 20 min, and the absorbance was measured at 680 nm. In the control group, trichloroacetic acid was added to the substrate mixture before adding the enzyme, and the other conditions were the same. One unit of protease activity (U) was defined as the amount of enzyme required to liberate 1 μg of tyrosine per minute under the assay conditions (pH 7.0).

Protein content was quantified using a BCA protein assay kit (Beyotime Biotechnology, Shanghai, China) with bovine serum albumin (BSA) as the standard [9].

### 2.5. Purification of Proteases

The protease purification process was conducted following the method outlined by Sun et al. [10] with certain modifications. Initially, the crude enzyme mixture was subjected to ammonium sulfate precipitation. The protease was precipitated using ammonium sulfate to 80% saturation and allowed to settle at 4 °C for 2 h. The precipitate was then collected by centrifugation at 6000 rpm for 10 min at 4 °C. The collected precipitate was resuspended in Tris-HCl buffer (20 mM, pH 7.0), and the resulting solution was dialyzed against a 10 kDa dialysis bag for 36 h at 4 °C. The dialysate was subsequently lyophilized and stored at −80 °C. The lyophilized material was reconstituted in phosphate buffer (20 mM, pH 7.0) and concentrated at 10 mL using a 3 kDa concentrator tube (MilliporeSigma, Burlington, MA, USA).

The concentrated solution was then applied to a DEAE-Sepharose FF column (1 cm × 5 cm) equilibrated with Tris-HCl buffer (20 mM, pH 7.0). The sample was passed through a 0.45 μm microporous membrane and eluted with a 1 M NaCl solution at a 1.0 mL/min flow rate. Linear gradient elution was performed using 0.2, 0.6, and 1 M NaCl solutions, and the resulting protein absorption peaks were analyzed for protein content and enzyme activity.

Finally, the major absorption peaks from the ion-exchange column were applied to a Superdex 75 10/300 (1 cm × 12.5 cm) gel filtration column equilibrated with 20 mM phosphate buffer (pH 7.0) and 0.1 M NaCl. The protease fraction was eluted at a flow rate of 0.3 mL/min, and each protein absorption peak’s protein content and enzyme activity were determined to identify the major active peaks.

### 2.6. Sodium Dodecyl Sulfate-Polyacrylamide Gel Electrophoresis (SDS-PAGE)

To estimate the molecular weight of the protease at various purification stages, non-reducing SDS-PAGE analyses were conducted using 12% acrylamide separating gels and 4% acrylamide concentrating gels. The concentrated gels were prepared at 80 V, and the separating gels were run at 120 V. After electrophoresis, the gels were stained with Coomassie Brilliant Blue R250 for 1 h and subsequently destained to observe the gel patterns. The molecular weights of the destination bands were estimated using standard proteins.

### 2.7. Characterization of Proteases

#### 2.7.1. Effects of pH and Temperature on the Activity and Stability of Protease

The influence of pH on enzyme activity was investigated using casein as the substrate across a pH range of 4.0–9.0. The buffers utilized were 0.05 M acetic acid–sodium acetate buffer (pH 4.0–5.0), 0.05 M sodium dihydrogen phosphate–disodium hydrogen phosphate buffer (pH 6.0–7.0), and 0.05 M Tris-HCl buffer (pH 8.0–9.0).The pH of the sample enzyme solution was adjusted with the different pH buffer systems mentioned above, and the sample enzyme solution was made to react with casein in the different pH buffer systems, placed in a 40 °C water bath and held for 30 min, and then removed and cooled at 4 °C, and then the residual enzyme activity was determined by the method in Section 2.4, and the enzyme activity was determined with the untreated sample enzyme solution at the optimum pH as a control, and the maximum enzyme activity was expressed as 100%, and the other enzyme activities were expressed as relative enzyme activity. The other enzyme activities were expressed as relative enzyme activities, so as to determine the optimum pH for the enzyme activity reaction.

The optimum reaction temperature was determined by determining the enzyme activity of the samples at optimum ph at different reaction temperatures (20, 30, 40, 50, 60 and 70 °C). The sample enzyme solution was reacted with the substrate at optimum pH and near optimum temperature (20, 30, 40, 50 and 60 °C) for 0, 20, 40, 60, 80 and 100 min, and then the enzyme activity of the sample was determined by expressing the maximum enzyme activity as 100% and the other enzyme activities as relative enzyme activities.

#### 2.7.2. Effects of Metal Ions on Enzyme Activity

The impacts of Ca^2+^, Cu^2+^, Fe^2+^, Mg^2+^, Zn^2+^, Ni^2+^, and Co^2+^ on enzyme activity were evaluated. Under optimal reaction conditions for the sample enzyme solution, the sample was mixed with different metal-ion stock solutions (CaCl_2_, NiCl_2_, CoCl_2_, CuSO_4_, FeSO_4_, ZnSO_4_, MgSO_4_) to achieve a final concentration of 5 mM of each metal ion. The corresponding enzyme activity was then determined, and the enzyme activity of the sample mixture, which was unaffected by metal ions, was set at 100%.

#### 2.7.3. Effects of Chemical Modulators on *Volvariella volvacea* Neutral Protease

The protease inhibitors including phenylmethylsulfonyl fluoride (PMSF), β-mercaptoethanol, and ethylenediaminetetraacetic acid (EDTA), were mixed with the sample enzyme mixture at final concentrations of 1 mM, 2 mM, 4 mM, and 10 mM, respectively. The surfactants included cetyltrimethylammonium bromide (CTAB), Tween-80, and Triton X-100, which were mixed with the sample enzyme solution at a final concentration of 5% (*w*/*v*). The enzymes were then incubated at the optimal temperature and pH for 10 min, and the corresponding enzyme activity was determined and set to 100% for untreated enzyme activity.

#### 2.7.4. Calculation of Kinetic Parameters of Enzymatic Reactions

The kinetic parameters Vmax and Km were determined using Lineweaver–Burk plots. Casein was used as the substrate at concentrations of 0, 1, 2, 6, 10, and 14 g/L. The substrate solutions were mixed with the enzyme mixture and incubated at pH 7.0 and 40 °C for 10 min. Enzyme activity was then measured according to the method described in Section 2.4.

### 2.8. Hydrolytic Action of Volvariella volvacea Protease on Isolated Soybean Proteins

#### 2.8.1. Determination of Hydrolysis

A soybean-isolate protein solution at a specific pH was prepared, and the enzyme was added for a designated period. After the enzyme digestion, the pH was adjusted to the initial substrate pH using 0.5 mol/L sodium hydroxide, and the volume of sodium hydroxide consumed was recorded. The degree of hydrolysis was calculated using the following formula:

DH (%) = B × N/(α × Mp × hot)

where B is the volume of sodium hydroxide consumed, N is the concentration of sodium hydroxide, α is the average degree of dissociation of soy isolate protein (α = 0.44 at pH 7), Mp is the mass of hydrolyzed protein, hot is the number of millimoles of peptide bonds per gram of protein substrate; hot (soy isolate protein) = 7.8 mmol/g.

#### 2.8.2. Effects of pH and Temperature on Proteolytic Cleavage of Soybean Isolates by Proteases

The soybean-isolate protein was prepared as a 5% (*w*/*v*) solution in 50 mM phosphate buffer and adjusted to pH values of 6.0, 6.5, 7.0, 7.5, and 8.0. The solutions were then heated in a water bath at 85 °C for 15 min to denature the protein. The purified protease was added to the soybean-isolate protein solutions at an enzyme-to-substrate ratio of 100 U/g. The reaction mixtures were then oscillated on an orbital shaker (150 rpm) at 50 °C for 3 h. After the reaction, the mixtures were placed in a boiling water bath for 15 min to inactivate the enzyme. The degree of hydrolysis was then determined.

To assess the effect of temperature on enzymatic hydrolysis, soybean-isolate protein was prepared as a 5% (*w*/*v*) solution at pH 7.0. The hydrolysis reaction was then carried out at different temperatures (35 °C, 40 °C, 45 °C, 50 °C, and 55 °C) with shaking (150 rpm) for 3 h. The degree of hydrolysis of the soybean-isolate protein by the *V. volvacea* protease was determined at each temperature.

#### 2.8.3. Effect of Enzyme Digestion Time on the Protease Digestion of Soybean Isolates

The purified protease and trypsin solutions were mixed separately with the soybean-isolate protein mixture (enzyme-to-substrate ratio of 100 U/g). The reaction mixtures were oscillated on a single orbital shaker (150 rpm) for various durations (1 h, 2 h, 3 h, 4 h, and 5 h) under their respective optimal conditions: *Volvariella volvacea* protease (pH 7.0, 50 °C) and trypsin (pH 8.0, 37 °C). The degree of hydrolysis of the soybean-isolate protein by the *Volvariella volvacea* protease and trypsin was then determined. Finally, the mixtures were centrifuged at 8000 rpm for 10 min, and the supernatants were collected and stored for further analysis.

#### 2.8.4. Determination of Peptide Mass Distribution and Free Amino Acid Content in Enzyme Digestion Products

The peptide mass distribution in the enzyme digestion products was determined by high-performance liquid chromatography (HPLC). The analysis used a Waters BEH SEC column with a mobile phase of 50 mM disodium phosphate and 50 mM sodium dihydrogen phosphate. Detection was carried out using an ultraviolet (UV) detector at a wavelength of 220 nm. The injection volume was 2 μL, and the column temperature was maintained at room temperature.

For free-amino-acid determination, the enzymatic digestion samples (from Section 2.8.3) underwent pretreatment as follows: A 2 mL aliquot of the sample was mixed with 0.5 mL of 0.1 mol/L HCl containing 10% trichloroacetic acid (TCA). This mixture was then sonicated for 15 min to precipitate proteins. Following sonication, the mixture was centrifuged, and the resulting supernatant was collected for subsequent free-amino-acid analysis using an amino acid analyzer (Hitachi, Tokyo, Japan).

### 2.9. Statistical Analysis

All data are expressed as means ± standard deviations (means ± SD). Statistical analysis was performed using one-way ANOVA via SPSS 25.0. *p* < 0.05 was considered significant.

## 3. Results and Discussion

### 3.1. Protease Purification

**Figure 1:**
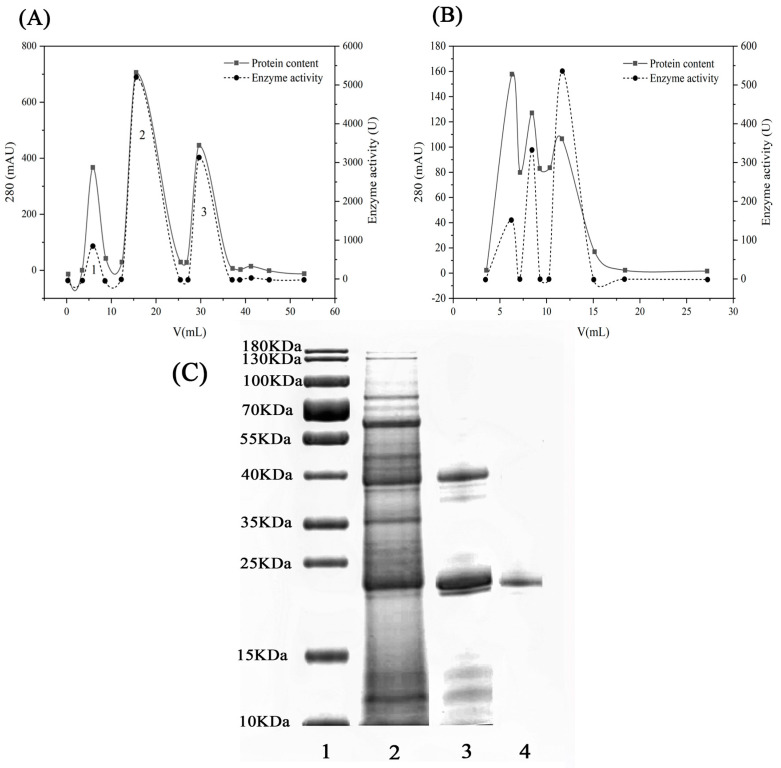
(**A**) Elution profile from DEAE-Sepharose FF. Numbers 1, 2, and 3 indicate distinct protein peaks; (**B**) Sephadex G-75 gel column chromatography profile. The protein content was expressed as the absorbance at 280 nm, and protease activity was expressed as the absorbance at 680 nm. (**C**) SDS-PAGE diagram of *V. volvacea* proteases. Lane 1, molecular weight marker; Lane 2, ammonium sulfate precipitation; Lane 3, DEAE FF ion-exchange chromatography; Lane 4, Sephadex G-75.

**Figure 2:**
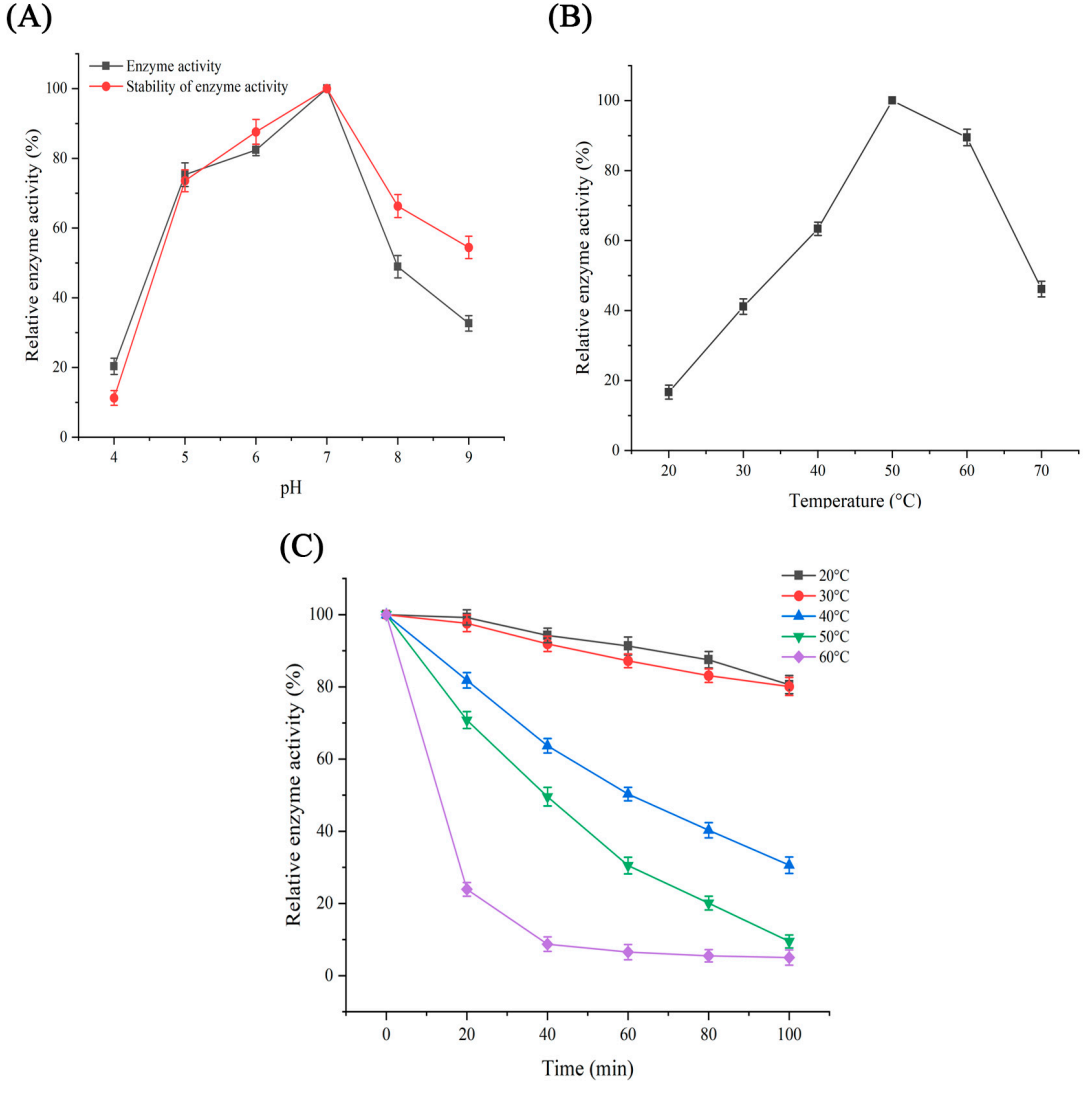
(**A**) Effects of pH on protease activity and stability; (**B**) effects of temperature on protease activity; (**C**) effects of temperature on protease stability.

The total enzyme activity (calculated as the sum of protease activity across all fractions in the ammonium sulfate precipitation step, based on the initial crude extract volume and measured enzyme activity) of the crude enzyme, after precipitation with 80% ammonium sulfate saturation, was calculated to be 7544 U (rounded to the nearest integer for clarity). Figure 1A,B shows the purification curves for ion-exchange chromatography and gel filtration. The protease mixture from *V. volvacea* was subjected to anionic column chromatography (DEAE FF) at 4 °C, and three distinct protease activity peaks were identified, as shown in Figure 2A. Among these peaks, the third absorption peak, D3, was the major protease absorption peak. The D3 fraction was further purified after ion-exchange chromatography using a gel column (Superdex 75 10/300). Figure 2B depicts the gel chromatography results, where three main absorption peaks were separated from the D3 fraction. The third peak was ultimately identified as the active fraction peak, S3, was determined based on enzyme activity and protein content. The purification steps are summarized in Table 1, and the final specific enzyme activity after purification was 286.82 U/mg, with a purification factor of 13.48. A similar study purified the extracellular cold-activated protease from *Chryseobacterium polytrichastri* using DEAE Cellulose-52 and DEAE Sephadex G-50, achieving a specific activity of 250 U/mg and 66.5-fold purification [11]. In another study, aspartic protease extracted from the defatted viscera of sardine (*Sardinella aurita*) was sequentially subjected to Sephadex G-100 gel filtration, Mono-S cation-exchange chromatography, ultrafiltration, and Sephadex G-75 gel filtration. The aspartic protease was purified 9.47-fold, with a recovery of 23.3% and a specific activity of 28.41 U/mg [12].

The purified *V. volvacea* protease produced a band with a molecular weight of 24 kDa on SDS-PAGE gel (Figure 1C). This enzyme is similar to the fibrinolytic enzyme from *Agrocybe aegerita* [13], which both originate from mushroom substrates. Other mushroom proteases have molecular weights ranging from 17 to 100 kDa [13]. For example, a novel protease isolated from the oyster mushroom *Pleurotus sajor-caju* had an estimated molecular weight of 65 kDa [14], and a new protease from *Coprinopsis atramentaria* was a monoclinic protein with a molecular weight of 32 kDa [15].

### 3.2. Effects of pH and Temperature on the Activity and Stability of Purified Proteases

To analyze the pH- and temperature-dependent effects of the *V. volvacea* protease systematically, we compared its behavior with that of proteases from diverse biological sources (fungi, bacteria, and plants), focusing on their ecological niches and industrial relevance. This approach aligns with identifying enzymes with unique stability profiles for food industry applications.

In terms of pH activity and stability, when casein was used as the substrate, the purified protease exhibited activity across pH values of 4–9, with optimal activity at pH 7 (Figure 2A). The activity sharply decreased at pH 8 (51% loss), indicating sensitivity to alkaline conditions. Stability assays (30 min pre-incubation at various pH values) revealed >75% residual activity at pH 5–7, highlighting its robustness in mildly acidic to neutral environments (Figure 2A). To determine the effect of pH on this protease, it was systematically compared with other proteases. In comparison, acidic proteases isolated from *Melilotus indicus* leaves demonstrated significant stability between pH levels of 4.0 and 6.0 [16], emphasizing evolutionary divergence in pH tolerance. The optimal activity of *Bacillus subtilis* S1 was observed at pH 8, demonstrating a broad tolerance range from pH 8 to 11 [17]. Furthermore, the researchers identified two proteases: one from *Bacillus subtilis* KM5B and another from *Aspergillus flavus* KMAF1. The bacterial protease derived from *Bacillus subtilis* exhibited activity within the pH range of 6 to 8, with peak activity occurring at pH 8.0. In contrast, the fungal protease obtained from *Aspergillus flavus* displayed maximum enzyme activity at pH 7.0 and maintained higher activity within the pH range of 7 to 9 [18]. This comparative framework underscores that the *V. volvacea* protease occupies a unique niche among fungal enzymes, combining neutral pH activity with moderate alkaline sensitivity—a trait advantageous for food-processing applications where neutral conditions prevail.

Temperature is a critical factor influencing protease activity. When the temperature exceeds a certain threshold, denaturation and inactivation of the enzyme can occur. Consequently, we assessed the changes in protease activity across various temperatures (20–70 °C) to identify the optimal reaction temperature. The results are presented in Figure 2B, indicating that enzyme activity remained above 50% within the 40–60 °C range, peaking at 50 °C. Temperature stability was determined by assessing residual activity after incubation at 20–60 °C for 0–100 min, with measurements taken at 20 min intervals. Figure 2C reveals key insights into the enzyme’s thermal tolerance and industrial applicability. The protease exhibited excellent stability under mild temperature conditions (20–40 °C); its activity decreased significantly with increasing temperature (50–70 °C), with <30% activity occurring within 20 min at 60 °C. High temperatures (>50 °C) led to irreversible denaturation of the enzyme, whereas progressive inactivation at 40–50 °C may be related to conformational flexibility. This property suggested that the protease is suitable for food processing, requiring only mild heating, and that high temperatures allow rapid reaction termination, facilitating process control. This finding aligns with previous studies on proteases purified from *P. minor* seeds, exhibiting maximal activity at 50 °C [19]. After maintaining the temperature at 40 °C for one hour, the protease activity was approximately 50%, suggesting better stability below this temperature; however, after holding at 60 °C for twenty minutes, the enzyme activity decreased to approximately 20%. Similar observations were reported for proteases derived from the pyloric cecum of *Colossoma macropomumj*, which retained approximately 80% of their initial activity following thirty minutes of incubation at 40 °C [20].

### 3.3. Effects of Metal Ions on Protease Activity

Numerous studies have demonstrated that adding divalent metal ions can significantly influence protease activity [21]. Consequently, this study aimed to investigate the effects of various metal ions on protease activity. The effects of seven metal ions (5 mM final concentration) on *V. volvacea* protease activity were examined (Table 2). A concentration of 5 mM was selected based on prior studies to ensure sufficient interaction with the enzyme’s active site while avoiding nonspecific binding [13]. Cu^2+^ and Ni^2+^ strongly inhibited enzyme activity, likely due to their competition with substrate molecules at the enzyme’s active site, thereby diminishing the binding affinity between the enzyme and its substrate and subsequently affecting enzymatic activity. A novel fibrinolytic enzyme derived from the edible mushroom *Lyophyllum shimeji* [22] and a fibrinolytic enzyme obtained from the edible mushroom *Pleurotus ferulae* [23] also exhibited strong inhibition by Cu^2+^. In contrast, Mg^2+^, Co^2+^, and Fe^2+^ had a relatively low impact on protease activity, resulting in only weak inhibition. These metal ions are likely present at the enzyme’s catalytic site, potentially protecting against thermal inactivation due to their high activation levels. According to the relevant literature, Mn^2+^, Mg^2+^, and Ca^2+^ all help prevent the thermal denaturation of proteases [16]. Furthermore, Ca^2+^ significantly stimulated protease activity. Similarly, Ca^2+^ enhanced the activities of *A*. *oryzae* NRRL 221 protease [24] as well as the gamma-Proteobacterium spp. protease [25]. However, it is essential to note that the mechanism underlying Zn^2+^ inhibition might differ from those related to other metal ions, as most neutral proteases contain Zn^2+^ within their structural centers. Following the addition of exogenous Zn^2+^ at elevated concentrations, an apparent inhibition of protease activity was observed [26].

### 3.4. Effects of Protease Inhibitors and Surfactants on Enzyme Activity

As a protein with catalytic properties, introducing inhibitors and organic reagents during its catalytic process can diminish enzyme activity or lead to complete inactivation. Investigating the effects of these reagents on proteases is a fundamental approach to studying their structural characteristics and the functional groups present at their active sites. This methodology is essential for elucidating the catalytic and metabolic mechanisms underlying protease function [27].

Protease inhibitors and surfactants were tested at concentrations optimized through preliminary assays (Table 3). Surfactant concentrations (e.g., 10 mM CTAB, 5% Tween-80) were chosen to reflect industrial processing conditions and align with prior studies on fungal proteases [28]. Among these, PMSF demonstrated a certain degree of inhibition of the protease; however, β-mercaptoethanol exhibited a more pronounced inhibitory effect. β-Mercaptoethanol can expose the sulfhydryl groups within the protease, thereby reducing disulfide bond formation and compromising its spatial structure’s stability, subsequently affecting its enzymatic activity [29]. These findings suggest that sulfhydryl groups are present in the structure of the protease. Conversely, the chelating agent EDTA appeared to have a minimal effect on enzyme activity, indicating either that the protease may not be classified as a metalloproteinase or that the concentration of EDTA used was insufficient to elicit a significant inhibitory effect. In addition to protease inhibitors, surfactants also exert considerable influence on enzyme activity. The *V. volvacea* protease retained 64% of its enzymatic activity when exposed to a 10 mM solution of the cationic surfactant CTAB, suggesting potential applications in detergent formulations. However, this enzyme displayed heightened sensitivity toward both Tween-80 and trilactone, which poses challenges for its use alongside nondiscretionary surfactants. Furthermore, alkaline proteases derived from *Bacillus altitudinis* W3 are activated by Ca^2+^ but are strongly inhibited by Mg^2+^ as well as by EDTA and PMSF [30].

### 3.5. Quantification of Kinetic Constants for Chemical Reactions

**Figure 3:**
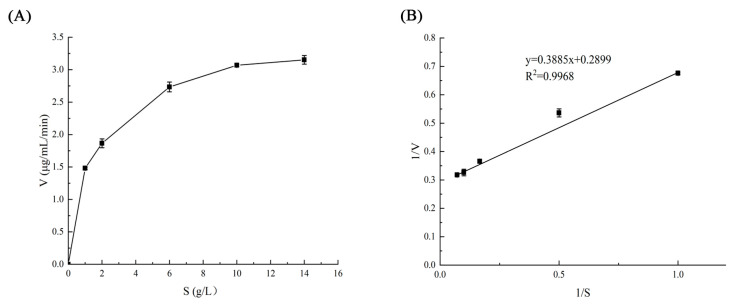
Kinetic parameters of *V. volvacea* protease using casein as the substrate. (**A**) Reaction rate (V) as a function of casein concentration (S). (**B**) Lineweaver–Burk plot (1/V vs. 1/S) for determining Km and Vm.

In kinetic studies, the rates of enzymatic reactions and their relationships with influencing factors are often examined through enzymatic reaction kinetics. This approach aims to identify optimal enzyme catalytic conditions and to elucidate the role of enzymes in metabolic processes. The protease activity at various substrate (casein) concentrations was assessed via the Folin method, where tyrosine production served as the vertical coordinate and substrate concentration as the horizontal coordinate, as illustrated in Figure 3A, where S represents substrate concentration (g/L) and V denotes reaction rate (μg/(mL·min)). This figure clearly shows that when S < 1 g/L, there is a linear relationship between the substrate concentration and the reaction rate. As S increases to 2 g/L, both V and S exhibit positive correlations indicative of first-order kinetics. With further increases in substrate concentration (S < 10 g/L), V becomes associated with the quantity of complex products formed by the interaction between the protease and substrate, marking this phase as mixed-order kinetics. Once the substrate concentration reaches a certain threshold (S > 10 g/L), interactions between the protease and substrate begin to stabilize due to saturation effects–where both the enzyme and substrate are fully engaged in binding. At this point, the reaction rate achieves its maximum value (Vm). Beyond this stage, V no longer correlates with S, characterizing this behavior as zero-order kinetics.

The double reciprocal plot for protease activity is presented as 1/V on the vertical axis against 1/S on the horizontal axis. According to our calculations from Figure 3B, we derive a material equation: 1/V = 0.3885 × (1/S) + 0.2899. From these computations, we can determine that the material constant Km = 1.34 g/L and Vm = 3.45 μg/(mL·min).

### 3.6. Unraveling the Enzymatic Potential of Volvariella volvacea Protease on Soybean Protein Isolates

#### 3.6.1. Effects of Optimum Hydrolysis pH, Temperature, and Enzymatic Time on Soybean-Isolate Proteins

**Figure 4:**
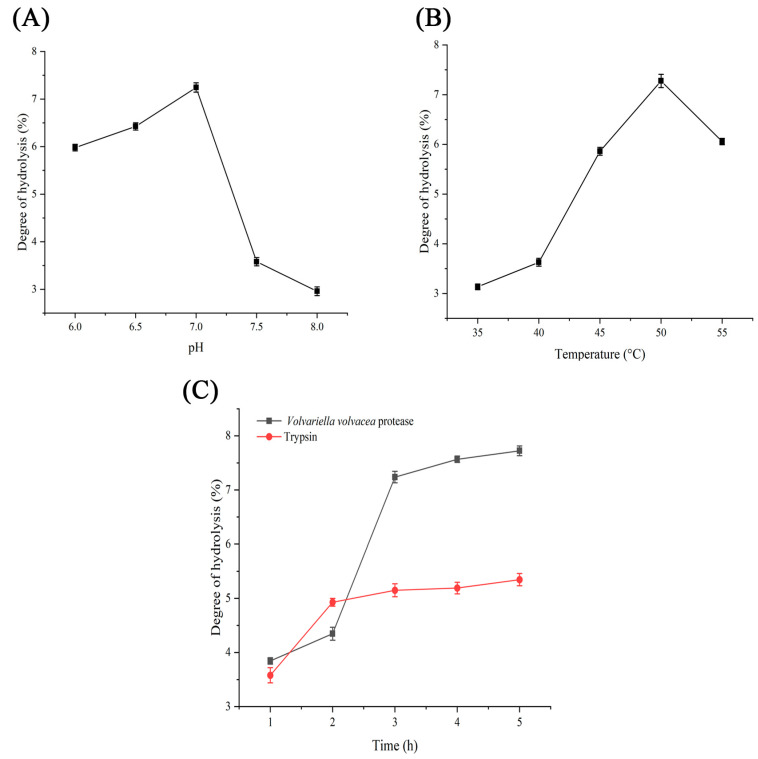
Enzymatic hydrolysis of soybean-isolate proteins by proteases. (**A**) Influence of pH on the degree of hydrolysis mediated by *V. volvacea* protease. (**B**) Impact of temperature on the degree of hydrolysis facilitated by *V. volvacea* protease. (**C**) Effects of reaction time on the degree of hydrolysis achieved with both proteases.

The optimal reaction pH and temperature for the proteases when casein was employed as the substrate were discussed previously. However, these parameters may be influenced by changes in the substrate used. Therefore, this section focuses on determining the optimum pH and temperature for the enzymatic hydrolysis of soybean-isolate proteins. As illustrated in Figure 4A, the optimal enzymatic pH was found to be 7, which is consistent with findings from experiments utilizing casein as a substrate. Moreover, this enzyme exhibited sensitivity under weakly alkaline conditions. In terms of enzymatic temperature, the optimum temperature for enzyme activity was 50 °C (Figure 4B), which again aligns with results obtained when casein was used as a substrate. Consequently, the ideal conditions for enzymatic hydrolysis are a pH of 7 and a temperature of 50 °C.

Under these optimal conditions of enzyme-digestion temperature and pH, the influence of enzyme-digestion time on the proteolytic process was examined. The degree of hydrolysis of soybean-isolate protein by *V. volvacea* protease and trypsin was compared. The results are illustrated in Figure 4C. In the initial stages of enzymatic hydrolysis, trypsin exhibited the highest degree of hydrolysis (4.95%). As time progressed, the interaction between the soybean-isolate proteins and proteases intensified until saturation was reached. At this point, the degree of hydrolysis achieved with the *V. volvacea* protease significantly surpassed that achieved with trypsin, reaching 7.58%, whereas the degree of hydrolysis achieved with trypsin was 5.24%.

#### 3.6.2. Peptide Distribution of Enzymatic Products

**Figure 5:**
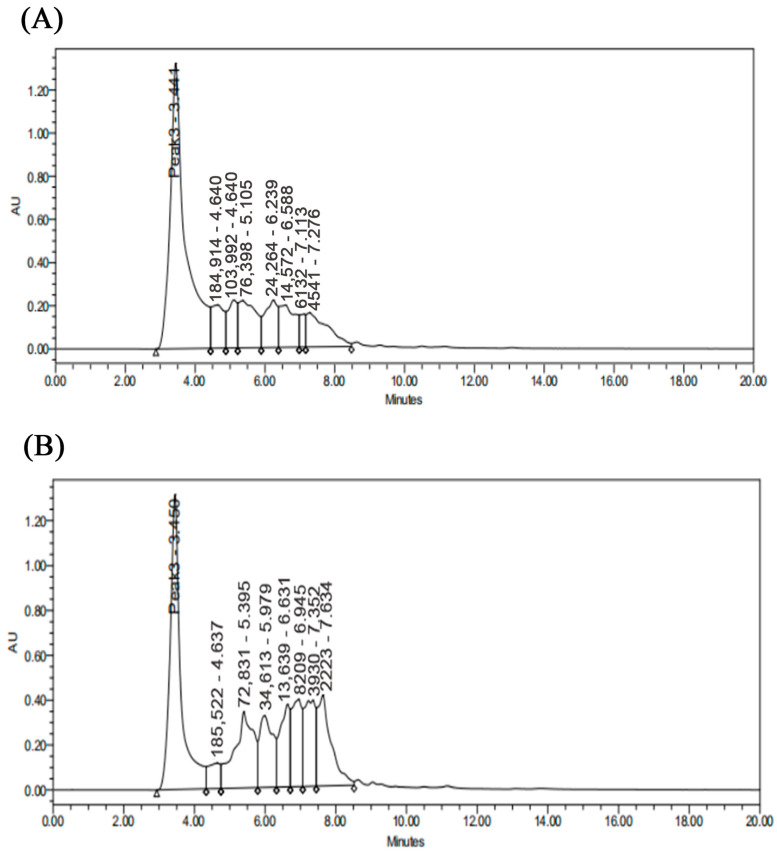
High-performance liquid chromatographic profile of the product following enzymatic digestion. (**A**) *V. volvacea* protease; (**B**) Trypsin.

The primary factors influencing enzymatic product properties are the molecular weight and distribution of their peptide components. Under optimal reaction conditions, soybean-isolate proteins were subjected to digestion with *V. volvacea* protease or trypsin. The resulting peak profiles were obtained through high-performance liquid chromatography (HPLC) (Figure 5). Notably, the two enzymes exhibited distinct enzymatic effects on soybean-isolate proteins (Table 4).

#### 3.6.3. Free Amino Acids in Enzymatic Products

The composition of free amino acids present in the products resulting from the enzymatic hydrolysis of soybean-isolate proteins is detailed in Table 5, revealing variations in the percentages of different free amino acids within these enzymatic hydrolysis products. Among the amino acids identified, several possess medicinal properties applicable to the pharmaceutical industry. For example, leucine (Leu) can regulate insulin synthesis to lower blood glucose levels, while arginine (Arg) promotes lymphocyte proliferation and enhances immune function [31]. Notably, medicinal amino acids such as aspartic acid (Asp), leucine (Leu), methionine (Met), lysine (Lys), arginine (Arg), glutamic acid (Glu), phenylalanine (Phe), tyrosine (Tyr), and glycine (Gly) constituted a significant proportion of the enzyme-digested products derived from *V. volvacea* proteins compared with those obtained through trypsinization. Hydrophobic amino acids—including valine (Val), isoleucine (Ile), leucine (Leu), and alanine (Ala)—are critically associated with the bitter taste characteristic of proteolytic products. Specifically, increased hydrophobic amino acid content within a peptide correlates with heightened bitterness levels [32]. Therefore, the enzymatic degradation of peptides may serve to decrease hydrophobic amino acid concentrations and subsequently reduce bitterness. The percentage of free hydrophobic amino acids in the trypsin digest was 29.32%, surpassing the 21.18% observed in the *V. volvacea* protease digest. These findings indicate that trypsin is more effective than *V. volvacea* protease in mitigating protein polypeptide bitterness. Furthermore, the ratio of fresh amino acids—namely, aspartic acid (Asp), glutamic acid (Glu), lysine (Lys), and glycine (Gly)—to total free amino acids revealed that the *V. volvacea* protease contained 18.57% fresh amino acids compared to 24.85% in the trypsin digests. These findings suggest a significantly greater presence of phenylalanine (Phe) and arginine (Arg) within the enzymatic products generated by the *V. volvacea* protease relative to other types of amino acids, indicating that its active site is enriched with Phe and Arg residues.

## 4. Conclusions

For the first time, a novel protease was successfully extracted and purified from the fruiting bodies of *Volvariella volvacea* subjected to cold treatment (4 °C). Through a multistep purification process involving ammonium sulfate precipitation, ultrafiltration, DEAE ion-exchange chromatography, and Superdex 75 gel filtration, the protease was obtained with a 13.48-fold purification and a specific activity of 286.82 U/mg. SDS-PAGE confirmed its homogeneity, revealing a molecular weight of approximately 24 kDa. Biochemical characterization classified the enzyme as a neutral protease, which displayed optimal activity at pH 7 and 50 °C. Notably, the enzyme exhibited sensitivity to alkaline conditions and thermal instability above 50 °C, while Ca^2+^ significantly enhanced its activity, and Ni^2+^/Cu^2+^ strongly inhibited it. Kinetic analysis yielded a Km of 1.34 g/L and a Vmax of 3.45 μg/(mL·min), indicating moderate substrate affinity comparable to other fungal proteases. The unique inhibition profile–marked by strong suppression from β-mercaptoethanol and differential responses to surfactants–suggests catalytic mechanisms distinct from bacterial or animal proteases. These findings expand the biochemical diversity of fungal proteases and provide a foundation for future structural studies to elucidate the role of sulfhydryl groups and metal ion interactions in their active sites. The protease effectively hydrolyzed soybean-isolate protein under mild conditions (pH 7, 50 °C), generating peptide profiles and free-amino-acid yields comparable to trypsin. This makes it a promising candidate for food industry applications, such as plant-based protein modification, bioactive peptide production, or meat tenderization, where neutral pH and moderate temperatures are preferred. Furthermore, its instability under alkaline conditions aligns with its potential role in addressing autolysis-related spoilage in *V. volvacea*, offering a biochemical target for extending postharvest shelf-life. While industrial detergents remain dominated by alkaline bacterial proteases, the cold-adapted nature and surfactant sensitivity of this enzyme warrant exploration in specialized biocatalytic niches, such as low-temperature food processing or environmentally friendly leather treatment. Subsequent studies should focus on structural characterization, genetic engineering to increase thermal stability, and the in vivo validation of its role in mushroom autolysis mechanisms.

## Figures and Tables

**Table 1:** Purification steps of the protease from *V. volvacea*.

Purification Steps	Protein Content (mg)	Total Activity (U)	Specific Enzyme Activity (U/mg)	Purification Factor
Ammonium sulfate precipitation	354.63 ± 12.42 ^a^	7544 ± 253 ^a^	21.27 ± 0.04 ^c^	1
DEAE-Sepharose FF	20.58 ± 1.26 ^b^	3186 ± 118 ^b^	154.84 ± 3.73 ^b^	7.28 ± 0.17
Sephadex-G75 Chromatography	1.88 ± 0.19 ^c^	539 ± 23 ^c^	286.82 ± 16.77 ^a^	13.48 ± 0.06

Note: Different letters in the same column indicate significant differences (*p* < 0.05).

**Table 2:** Effects of metal ions on enzyme activity.

Metal Ions	Concentration	Relative Activity (%)
None	-	100 ± 1
Ca^2+^	5 mM	150 ± 2
Cu^2+^	5 mM	43 ± 2
Fe^2+^	5 mM	89 ± 1
Mg^2+^	5 mM	96 ± 3
Ni^2+^	5 mM	36 ± 2
Co^2+^	5 mM	86 ± 2
Zn^2+^	5 mM	79 ± 3

**Table 3:** Effects of proteases modulated by protease inhibitors in the presence of certain detergents.

Chemical Reagents	Concentration	Relative Activity (%)
None	-	100 ± 2
PMSF	1 mM	64 ± 2
EDTA	4 mM	80 ± 1
β-mercaptoethanol	2 mM	38 ± 3
CTAB	10 mM	64 ± 2
Tween-80	5%	49 ± 2
Triton X-100	5%	27 ± 3

**Table 4:** Peptide content and distribution of enzymatic products.

Enzymatic Products	≥30 kDa (%)	10–30 kDa (%)	4–10 kDa (%)	≤4 kDa (%)
*V. volvacea* protease digestion products	73.92	15.09	2.12	8.86
Trypsin digestion products	60.2	8.2	18.84	12.76

**Table 5:** Proportion of free amino acids in proteolytic products generated by protease enzymes.

Free Amino Acid Species	*V. volvacea* Protease Digestion Products (%)	Trypsin Digestion Products (%)
Asp	4.77	0.81
Thr	2.99	3.15
Ser	1.89	5.34
Glu	5.87	10.08
Gly	2.57	2.85
Ala	8.80	8.97
Val	2.84	4.12
Cys	-	0.54
Met	3.57	2.76
Ile	1.29	4.77
Leu	8.25	11.45
Tyr	4.68	9.92
Phe	18.32	14.45
Lys	5.35	11.11
His	2.00	3.77
Arg	20.15	-
Pro	2.51	0.45
Trp	4.13	5.44

Note: - indicates below detection limit.

## Data Availability

The original contributions presented in this study are included in the article. Further inquiries can be directed to the corresponding authors.
